# Vitamin K Epoxide Reductase Complex Subunit 1-Like 1 (VKORC1L1) Inhibition Induces a Proliferative and Pro-inflammatory Vascular Smooth Muscle Cell Phenotype

**DOI:** 10.3389/fcvm.2021.708946

**Published:** 2021-10-27

**Authors:** Adem Aksoy, Muntadher Al Zaidi, Elena Repges, Marc Ulrich Becher, Cornelius Müller, Johannes Oldenburg, Sebastian Zimmer, Georg Nickenig, Vedat Tiyerili

**Affiliations:** ^1^Department of Cardiology, Heart Centre, University of Bonn, Bonn, Germany; ^2^Institute of Experimental Haematology and Transfusion Medicine, University of Bonn, Bonn, Germany

**Keywords:** vascular inflammation, vitamin K, oxidative stress, ER stress, vascular remodeling

## Abstract

**Background:** Vitamin K antagonists (VKA) are known to promote adverse cardiovascular remodeling. Contrarily, vitamin K supplementation has been discussed to decelerate cardiovascular disease. The recently described VKOR-isoenzyme Vitamin K epoxide reductase complex subunit 1-like 1 (VKORC1L1) is involved in vitamin K maintenance and exerts antioxidant properties. In this study, we sought to investigate the role of VKORC1L1 in neointima formation and on vascular smooth muscle cell (VSMC) function.

**Methods and Results:** Treatment of wild-type mice with Warfarin, a well-known VKA, increased maladaptive neointima formation after carotid artery injury. This was accompanied by reduced vascular mRNA expression of VKORC1L1. *In vitro*, Warfarin was found to reduce VKORC1L1 mRNA expression in VSMC. VKORC1L1-downregulation by siRNA promoted viability, migration and formation of reactive oxygen species. VKORC1L1 knockdown further increased expression of key markers of vascular inflammation (NFκB, IL-6). Additionally, downregulation of the endoplasmic reticulum (ER) membrane resident VKORC1L1 increased expression of the main ER Stress moderator, glucose-regulated protein 78 kDa (GRP78). Moreover, treatment with the ER Stress inducer tunicamycin promoted VKORC1L1, but not VKORC1 expression. Finally, we sought to investigate, if treatment with vitamin K can exert protective properties on VSMC. Thus, we examined effects of menaquinone-7 (MK7) on VSMC phenotype switch. MK7 treatment dose-dependently alleviated PDGF-induced proliferation and migration. In addition, we detected a reduction in expression of inflammatory and ER Stress markers.

**Conclusion:** VKA treatment promotes neointima formation after carotid wire injury. In addition, VKA treatment reduces aortal VKORC1L1 mRNA expression. VKORC1L1 inhibition contributes to an adverse VSMC phenotype, while MK7 restores VSMC function. Thus, MK7 supplementation might be a feasible therapeutic option to modulate vitamin K- and VKORC1L1-mediated vasculoprotection.

## Introduction

Oxidative stress and inflammation contribute to adverse cardiovascular remodeling, eventually resulting in atherosclerosis and vascular dysfunction (1). Vascular smooth muscle cells (VSMC) participate in atherosclerotic plaque growth in different ways. In response to atherosclerotic stimuli, VSMC may turn hyper-proliferative and promote neointima formation. Neointima formation is the main driver of in-stent restenosis and is further enhanced by pro-inflammatory stimuli in the vasculature (2, 3). The ability of VSMC to limit vascular inflammation and to preserve their physiological phenotype is essential to slow the progression of atherosclerosis and neointima formation in particular.

Vitamin K describes a group of fat-soluble vitamins that are required as co-factors for γ-carboxylation of proteins (4). Vitamin K1 and the K2 vitamins menaquione-4 (MK4) and menaquione-7 (MK7) are the best-known members of the vitamin K group. Among these, MK7 is the most potent K vitamin in human physiology (5). In the vitamin K cycle, vitamin K is recycled by reactions catalysed by vitamin K epoxide reductase complex subunit 1 (VKORC1) and its recently described isoenzyme, vitamin K epoxide reductase complex subunit 1-like 1 (VKORC1L1). These two enzymes serve as targets of vitamin K antagonists (VKA), such as Warfarin, that are used in therapeutic anticoagulation regimen (6, 7). Evidence has emerged that VKA also contribute to cardiovascular damage (8–10). In addition, dietary supplementation with vitamin K was shown to be a safe and feasible option to decelerate vascular disease (11–13). Furthermore, vitamin K provides anti-oxidative properties and acts as a potent free-radical scavenger (14, 15). Hence, elucidating the mechanism of VKA-induced vascular dysfunction and vitamin K-dependent vasculoprotection is of relevance. Compared to VKORC1, VKORC1L1 is expressed at lower levels in the liver, the place where coagulation factors are synthesised (16), and thus has a lower ability to γ-carboxylate the coagulation factors (7, 17). Upon oxidative stress, VKORC1L1 is upregulated, while VKORC1 is downregulated (18). Furthermore, VKORC1L1-knockout cells are far more sensitive to oxidative stress as compared to VKORC1-knockout cells (19). Thus far, there is no data regarding the significance of VKORC1L1 in cardiovascular diseases, although a recent mRNA expression analysis described VKORC1L1 as a putative oxidative-stress-related gene in coronary artery disease (20).

Both VKOR proteins are localized in the membrane of the endoplasmic reticulum (ER) (18). In the ER, proteins are folded under sensitive environmental conditions and at a precisely regulated redox state. Disturbances of these conditions constitute ER stress, a major contributor to vascular inflammation (21, 22). The VKOR system is known to interact with protein folding (23), but the role of VKOR proteins in ER stress remains unclear.

Therefore, the aim of this study was to analyse the role of VKORC1L1 in neointima formation and on VSMC inflammation and proliferation.

## Materials and Methods

### Animal Procedures and Diets

Animal experiments were performed in accordance with the animal protection law stated in the German civil code and the National Office for Nature, Environment and Consumer Protection in Recklinghausen, North Rhine-Westphalia (*Landesamt für Natur, Umwelt und Verbraucherschutz;* LANUV). We used six-week-old C57BL/6 wild-type mice (Charles River, Sulzfeld, Germany). Animals were maintained in a 22°C room with a 12 h light/dark cycle and received food and drinking water *ad libitum*. The surgical intervention was performed under a dissecting microscope (MZ6; Leica). For the carotid-injury procedure, mice were anesthetized with intraperitoneal injections of 150 mg/kg body weight ketamine hydrochloride (Ketanest, Riemser, Greifswald, Germany) and 0.1 mg/kg body weight xylazine hydrochloride (Ceva, Duesseldorf, Germany). Access to the carotid artery was obtained by performing a midline skin incision from directly below the mandible toward the sternum. Careful preparation of the left common carotid artery and carotid bifurcation was performed. Two filaments were placed in the proximal and distal segments of the external carotid artery, and the distal ligature was then pinched with clamps. The internal and common carotid arteries were temporarily occluded to perform a transverse arteriotomy between the ligatures of the external carotid artery and to insert a flexible wire (0.13 mm in diameter), that is slightly curved (30°) at the tip and completely fills out the vessel. For endothelial denudation, a pullback of the wire was performed in a rotating manner for five times per animal and then removed. Then the external carotid artery was closed below the site of puncture with a ligature, and the blood flow of common and internal carotid artery was released. The skin was then sutured. The mice were postoperatively allowed to recover individually. During recovery period, animals were held in a warm environment by use of water-circulating heat pads and were closely observed. Food and water intake were monitored. The recovery period was four h long. Full recovery was confirmed by return of righting reflex and stable respirations. Thereafter, the animals were returned to their littermates and randomized to three different diet groups. Group A received vehicle, group B received a vitamin K1 (1.5 mg/g food)-enriched diet, and group C received a vitamin K1 (1.5 mg/g food) and warfarin (2 mg/g food)-enriched diet. The composition of the food regimes was adapted according to Schurgers et al. (9) After 14 days, the mice were sacrificed. All mice were anesthetized using 2% isoflurane and an intraperitoneal injection of fentanyl (0.05 mg/kg) and midazolam (5 mg/kg), then euthanized by cervical dislocation. All tissue and blood samples were collected and processed immediately after the mice were sacrificed.

For factor x activity measurement, blood samples (100 μl) were collected, immediately added onto citrate-containing tubes in a ratio of 10:1 (blood/citrate) and mixed. After centrifugation, the resulting plasma was diluted in factor x deficient plasma. This step is necessary to achieve that the factor x activity is the limiting factor in the following thromboplastin time measurement. Thromboplastin time corresponding to factor x acticity was then measured and normalized as percentage to a previously determined normal plasma. Factor X activity was measured at the Institute of Experimental Haematology and Transfusion Medicine at the University Hospital of Bonn, Germany.

Vitamin K1 and warfarin were purchased from Sigma-Aldrich (Cat# 47773 and A2250) and provided to Ssniff-Spezialdiäten, Soest, Germany. Ssniff-Spezialdiäten then mixed vitamin K1 or vitamin K1 and warfarin into the regular mice diet.

### Cell Culture

Primary human coronary artery smooth muscle cells (HCASMC) were purchased (PromoCell, Germany; Cat# C-12511) and cultured in their respective cell medium (PromoCell, Cat# C-22062), using standard conditions (37°C, 5% CO_2_, 100% relative humidity). Once the cells reached 70–90% confluence, the medium was removed, and cells were washed once before passaging by trypsinisation. Cells from passages 6–8 were used for experiments.

For siRNA transfection, the cells were transfected with HiPerFect Transfection Reagent (Qiagen, Netherlands; Cat# 301705), using the Reverse Transfection Protocol. Briefly, HiPerFect plus an siRNA directed against *VKORC1L1* (Qiagen, # SI04138407) or a scrambled siRNA (Qiagen, # 1027280), not directed toward any mRNA, were diluted in serum-free media and mixed by vortexing. The transfection mixtures were incubated at room temperature for ten min and then transferred into empty cell-culture wells. In the meantime, cells were trypsinised and then seeded on top of the transfection complexes. The final siRNA concentration was 10 nM and 0.5% V/V for HiPerFect Transfection Reagent. Following transfection, the cells were incubated under the above-mentioned conditions for 46 h before a readout was taken.

### Viability Assay

alamarBlue^TM^ HS Cell Viability Reagent (Thermo Fisher Scientific, USA; Cat# A50100) was added onto pre-treated cells. The viability reagent and cell mixtures were incubated for 4 h under standard conditions (37°C, 5% CO_2_, 100% relative humidity) and protected from light. Then the absorbance was measured using an Infinite M200 Microplate Reader (Tecan, Switzerland).

### Western Blot

Cells were lysed with RIPA Buffer (Sigma-Aldrich, Cat# R0278) containing 1:25 Protease Inhibitor Cocktail (Roche, Cat# 4693132001). The lysates were centrifuged at 13,000 x g for 10 min at 4°C. Protein concentration in the supernatant was quantified by a Qubit Protein Assay (Thermo Fisher Scientific, Cat# Q33211) in a Qubit-4 Fluorometer (Thermo Fisher Scientific). 25 μg of the resulting protein were loaded onto an SDS-PAGE gel and electrophoresis was initiated on a Mini Protean system (Bio-Rad). Proteins were then transferred to a nitrocellulose membrane (Carl Roth, HP40.1) for western blotting. For blocking of the membrane, we used 5% BSA for one h at room temperature. Afterwards the membrane was incubated with the respective primary antibody overnight at 4°C.

The next morning, the membrane was washed three times with 0.1% tris-buffered saline containing 0.1% Tween 20, before addition of the secondary antibody. After an one-h long incubation at room temperature, the membrane was washed again three times before detection was performed with an ECL Western Blot Detection Reagent (Sigma-Aldrich, Cat# RPN2232) on a ChemoCam HR16-3200 Imager (Intas).

The following antibodies were used: VKORC1L1 (final concentration: 0.2μg/ml, Sigma-Aldrich, Cat# HPA053954), ß-actin (dilution 1:2000, Sigma-Aldrich, Cat# A1978), anti-mouse-IgG secondary antibody (dilution 1:10,000, Sigma-Aldrich, Cat# A9044) and anti-rabbit-IgG secondary antibody (dilution 1:10,000, Sigma-Aldrich Cat# A0545).

### Reactive Oxygen Species (ROS) Measurement

The formation of ROS was measured by using L-012 chemiluminescence and 2',7'-dichlorofluorescein diacetate (DCFDA, Sigma-Aldrich, USA; Cat# D6883) chemiluminescent assays. L-012, formed by derivation of luminol, has a high sensitivity for superoxide radicals and does not exert redox cycling itself. Chemiluminescence was determined over 15 min in a scintillation counter (Lumat LB 9501, Berthold) at one-min intervals.

For DCFDA assay, cells were seeded one day prior to the experiment unto a dark-bottomed 96-well microplate protected from light and were incubated at standard conditions. After 24 h, the cells were stimulated with 75 μM hydrogen peroxide (H_2_O_2_) for one h. Since H_2_O_2_ is a potent inducer of free radical formation (24), cells were stimulated with H_2_O_2_ to measure ROS formation in response to an oxidative stressor. Then, a dilution of 50 μM DCFDA was added for 45 min. Finally, the DCFDA solution was removed and the cells were washed before the addition of PBS. Fluorescence was detected immediately using a microplate reader at maximum excitation and emission of 492 nm and 527 nm, respectively as previously described (25, 26).

To specifically measure the source of ROS, we utilized an Amplex^TM^ Red Hydrogen Peroxide Assay Kit (Thermo Fisher Scientific, Cat# A22188). In presence of HRP, Amplex^TM^ Red reacts with H_2_O_2_ to a fluorescent product. Cells were seeded onto 12-well-plates and transfected with siRNA against VKORC1L1 or scrambled siRNA. After 46 h, 50 μl of the medium was collected and incubated for one h in the dark at 37°C with 50 μl of a mixture of HRP (0.1 U/ml) and Amplex^TM^ Red (50 μM). Thereafter fluorescency was measured in an Infinite M200 Microplate Reader (Tecan, Switzerland) microplate reader (excitation 560 nm, emission 590 nm).

### EdU Proliferation Assay

Cell proliferation was analysed using a Click-iT™ EdU Proliferation Assay for Microplates (Thermo Fisher Scientific, Cat# C10499). Briefly, the nucleoside analog EdU (5-ethynyl-2'-deoxyuridine) was added to live cells for 4 h. Then the cells were fixed and incubated with horseradish peroxidase (HRP), which becomes ligated to the DNA-incorporated EdU moiety by using click chemistry. Thereafter, Amplex^TM^ UltraRed, a reagent that is converted to a fluorescent product in presence of HRP, was added and the fluorescence was measured on a microplate reader at an excitation of 568 nm and an emission of 585 nm.

### Scratch Assay

Cultured cells were reverse transfected in 12-well plates as described above. After 48 h, cells were at confluence and a vertical scratch was created using a sterile pipette-tip (200 μl). Cells were washed and then cultured in reduced-serum medium (2.5%) to minimize proliferation without inducing extensive apoptosis. A specific position on each well was marked and images using an Axiovert 200M microscope (Carl Zeiss, Germany) were taken after 0, 4, 8, 12, and 24 h. Th area of cell migration into the scratched region was measured and calculated as a percentage of the initially scratched area.

### Quantitative Real-Time Polymerase Chain Reaction

Total RNA from the cells was isolated by using TRIzol^TM^ (Thermo Fisher Scientific, Cat# 15596018) and chloroform phase separation. The amount of RNA collected was quantified using a Nanodrop spectrophotometer (Nanodrop Technologies, USA). 0.5–2 μg of RNA were reverse transcribed by using an Omniscript RT Kit (Qiagen, Cat# 205113). Finally, quantitative real-time PCR was performed on a 7500 HT Real-Time PCR machine (Applied Biosystems, USA) using TaqMan gene expression assays (Thermo Fisher Scientific) and Gene Expression Master Mix (Thermo Fisher Scientific, Cat# 4369542). CT values up to 40 were used for analysis and all samples were run in triplicate. The values were analysed using the ΔΔCT method, by normalising to 18S ribosomal RNA.

The following TaqMan Assays were used: VKORC1L1 (Hs04989728_s1), VKORC1 (Hs00829655_s1), interleukin 6 (IL-6, Hs00174131_m1), nuclear factor ‘kappa-light-chain-enhancer' of activated B-cells (NF-κB, Hs00765730_m1), glucose-regulated protein 78 kDa(GRP78, Hs00607129_gH), C/EBP homologous protein (CHOP, Hs00358796_g1) and 18S ribosomal RNA (18S, Hs99999901_s1).

### Menaquinone-7 Supplementation Experiments

Cells were seeded into cell-culture wells and allowed to adhere. After 48 h, MK7 (Santa Cruz Biotechnology, USA; Cat# sc-218691) dissolved in DMSO (0.5 mg/ml) was mixed with fresh cell medium and added onto the cells. The incubation time was 24 h and the final MK7 concentrations in the medium ranged from 1 to 10μM. In some cases, cells were co-incubated with oxLDL (Oxidized low-density lipoprotein, Thermo Fisher Scientific, Cat# L34357), tunicamycin (Sigma-Aldrich, Cat# T7765) or platelet-derived growth factor (PDGF, Thermo Fisher Scientific; Cat# PHG0044).

### Statistical Analysis

Statistical analyses were performed using the software GraphPad Prism 9. Means of two groups were compared with an unpaired *t*-test. Means of more than two groups were compared by a one-way ANOVA followed by Bonferroni's multiple comparison test. The number of independent experiments as well as the applied tests for statistical significance are reported in the figure legends. All reported *p*-values are two- sided.

## Results

### Neointima Formation After Vascular Injury

**Figure 1:**
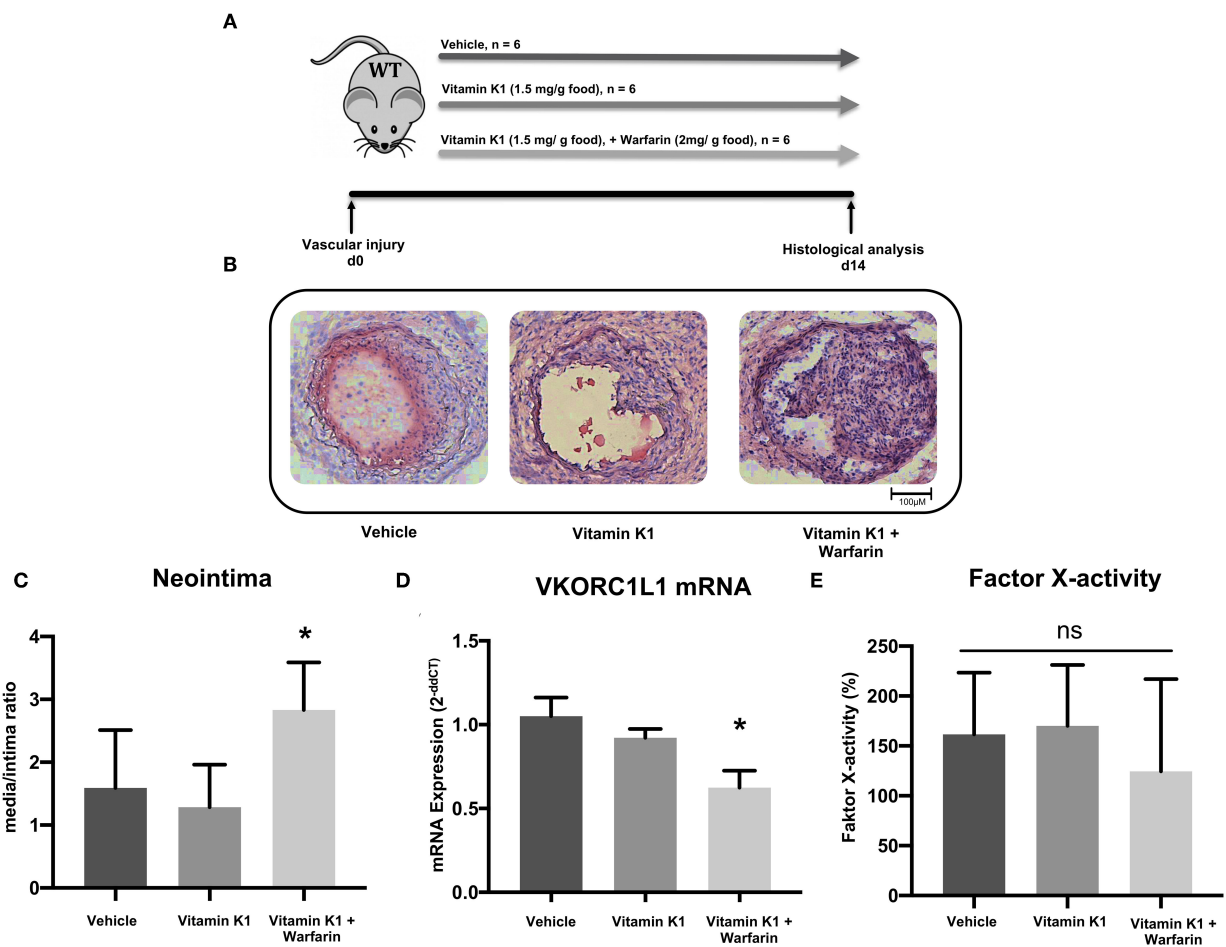
Vitamin K antagonism promotes vascular remodeling. **(A)** Schematic diagram of the design of the carotid artery injury experiment. After the procedure on 6–8 week-old C57Bl/6J wild-type mice, they were randomized in three groups that received either vehicle (group A), vitamin K1 (1.5 mg/g food)-enriched diet (group B), or vitamin K1 (1.5 mg/g food) and warfarin (2 mg/g food)-enriched diet (group C), *n* = 6 per group. **(B)** Images of neointima formation 14 days after carotid artery injury. Vessels were subjected to histological analysis by H.E. staining. **(C)** Quantitative assessment of neointima formation expressed as the media/intima ratio. **(D)** *VKORC1L1* mRNA expression in the abdominal aorta expressed as 2^−*ddCT*^ relative to control. **(E)** Factor X activity measured in citrate plasma samples after 14 days of treatment. Data are presented as the mean ± SEM; *p < 0.05; one-way ANOVA + Bonferroni's multiple comparison test for **(C-E)**.

In order to understand the possible role of VKORC1L1 in vascular remodeling, we utilised a murine vascular-injury model. C57/Bl6 mice were randomized to receive vehicle, a vitamin K1 (1.5 mg/g food)-enriched diet, or a vitamin K1 (1.5 mg/g) and warfarin (2 mg/g)-enriched diet after injury of the carotid artery (Figure 1A). Vitamin K1 was co-administrated with Warfarin in order to prevent the internal bleeding that is normally caused by warfarin and to assess warfarin's extrahepatic effect in isolation (27).

Mice fed a warfarin-enriched diet had increased neointima formation (media/intima ratio: 1.6 ± 0.9 vs. 1.3 ± 0.7 vs. 2.8 ± 0.7, *p* = 0.007, Figures 1B,C), and a significantly reduced VKORC1L1-mRNA expression in the abdominal aortic tissue (1.0 ± 0.1 vs. 0.9 ± 0.05 vs. 0.6 ± 0.1, *p* < 0.0001, Figure 1D). There was no significant difference between the three diet groups for coagulation activity, as assessed by a factor X activity assay (161.5 ± 61.9 % vs. 170.1 ± 61.0 % vs. 124.5 ± 92.5 %, *p* = 0.52, Figure 1E, control plasma: 100%).

### VKORC1L1 Regulates HCASMC Phenotype

**Figure 2:**
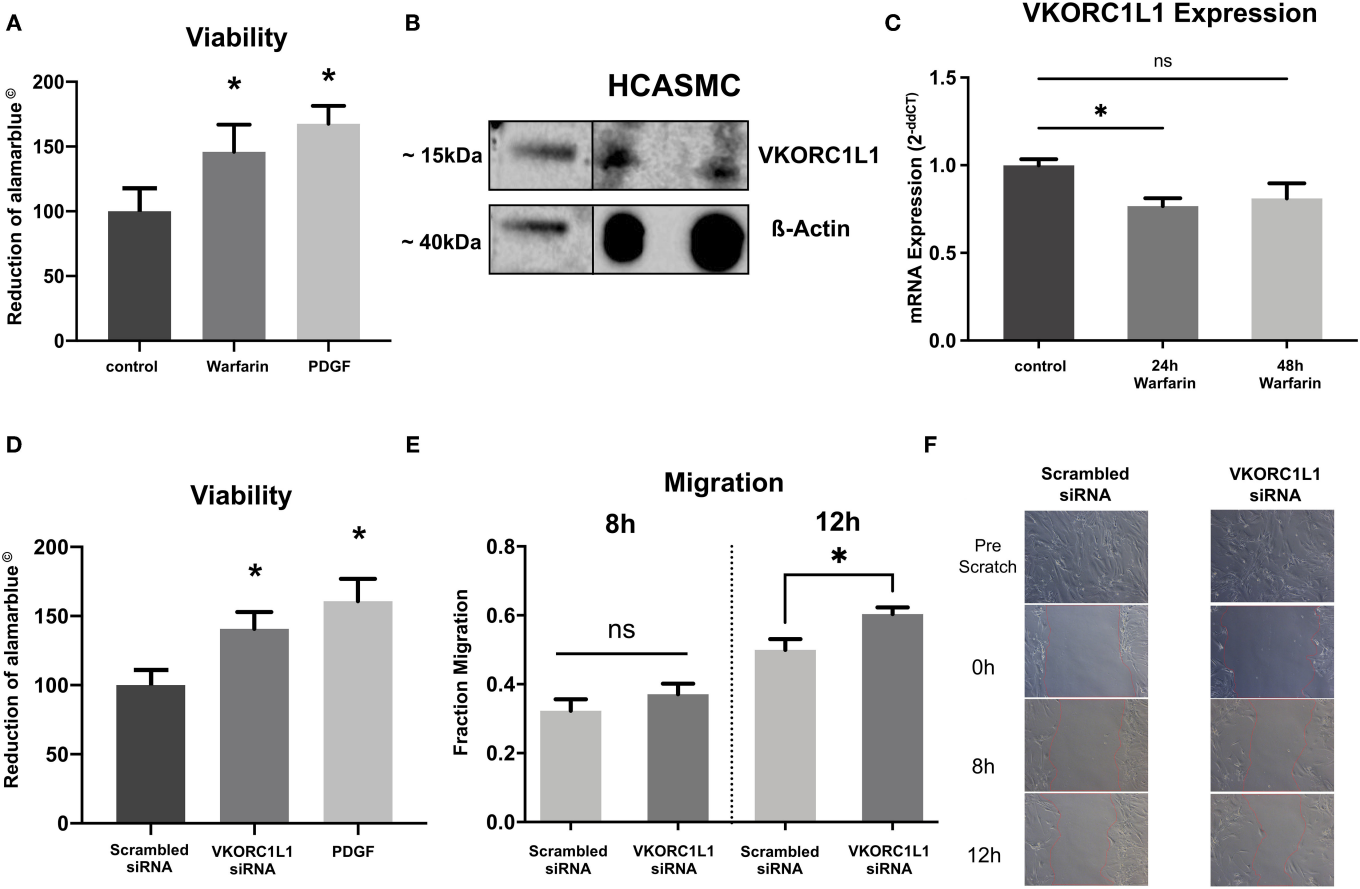
VKORC1L1-downregulation enhances HCASMC viability and migration. **(A**) Viability of HCASMC measured by alamarblue® assay after treatment with 1 μmol warfarin for 24 h. **(B)** VKORC1L1 protein expression in untreated HCASMC visualized by western blot. ß-Actin was used as housekeeping protein. **(C**) mRNA expression of VKORC1L1 in HCASMC after treatment with 1 μmol warfarin for 24 or 48 h. mRNA expression was measured by qPCR and quantified by the 2^−*ddCT*^ method. *n* = 5. **(D)** Viability of HCASMC measured by alamarblue® after transfection of siRNAs directed against *VKORC1L1* or a scrambled control. **(E)** Migration of siRNA-transfected HCASMC following a wound–scratch assay, measured by microscopic visualization after 8 and 12 hours. *n* = 7. **(F)** Representative pictures of migration. *p < 0.05. Data are presented as the mean ± SEM; *p < 0.05; one-way ANOVA + Bonferroni's multiple comparison test for **(A, C, D)**.

Neointima formation is mainly caused by hyperproliferation of vascular smooth muscle cells (2). Therefore, HCASMC were stimulated with warfarin (1 μM for 24 h). Stimulation with warfarin resulted in significantly higher viability in HCASMC compared to the control group (145.9 ± 21.1% vs. control, *p* = 0.003, Figure 2A).

Since this is the first study investigating the role of VKORC1L1 in vascular cells, we first performed western blot analyses to confirm expression of VKORC1L1. And indeed, VKORC1L1 is expressed significantly in HCASMC (Figure 2B).

Consistent to the *in vivo* experiments, we found warfarin treatment to reduce VKORC1L1 mRNA expression *in vitro* (1 μM, 24 h: 0.76-fold, *p* = 0.03 vs. control; 48 h: 0.81-fold, *p* = 0.07 vs. control; Figure 2C). To test possible specific effects of VKORC1L1, HCASMC were transfected with VKORC1L1-siRNA, which resulted in a largely reduced expression level of VKORC1L1 mRNA (Supplementary Figure 1). VKORC1L1 downregulated cells showed significantly higher viability compared to the control group (140.8 ± 12.1% vs. control, p = 0.0034, Figure 2D).

Migration of SMC is another major contributor to neointima formation. Therefore, we investigated the effect of VKORC1L1 inhibition on HCASMC migration *in vitro*. To address this point, wound–scratch assays were performed with transfected HCASMC, and the migration of cells was measured by microscopic visualization after 8 and 12 h. The migration of cells was not different between the VKORC1L1-siRNA transfected cells and the control group after 8 h (0.32 ± 0.09 vs. 0.37 ± 0.09 migration into the wound scratch, *p* = 0.3), whereas migration was enhanced significantly in knockdown cells compared to control cells after 12 h (0.49 ± 0.08 vs. 0.60 ± 0.05 migration into the wound scratch, *p* = 0.02, Figures 2E,F). After 24 h, migration into the scratch was fully achieved for both VKORC1L1-siRNA transfected cells and the control cell. Taken together, these data suggest, that VKORC1L1-downregulation enhances HCASMC viability and migration, both of which are crucial steps for neointima formation.

### VKORC1L1-Inhibition Promotes Reactive Oxygen Species (ROS) Formation

VKORC1L1 was found to exhibit anti-oxidative effects in human embryonic kidney cells. In those cells, VKORC1L1 deficiency was found to increase ROS formation (18, 19). Thus, we sought to investigate, if VKORC1L1 exhibits a similar role in HCASMC.

**Figure 3:**
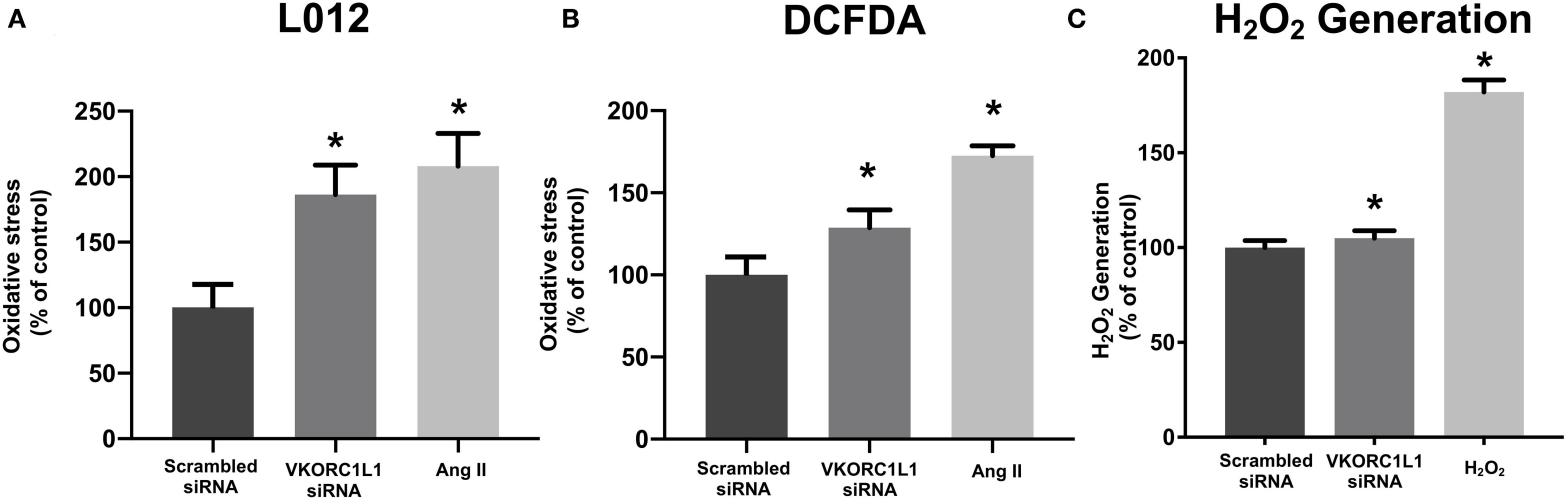
VKORC1L1-inhibition promotes reactive oxygen species formation. **(A, B)** ROS formation in HCASMC after transfection of siRNAs directed against *VKORC1L1* or a scrambled control. Measured by using L-012 **(A)** and DCFDA assays **(B)**. Angiotensin II (Ang II) was used as a positive control. n = 6–8. *p < 0.05 **(C)** H_2_O_2_ Generation in HCASMC was measured by an Amplex^TM^ Red assay after transfection with siRNA against VKORC1L1 or a scrambled control. Treatment with H_2_O_2_ was used as a positive control. *n* = 6. *p < 0.05. Data are presented as the mean ± SEM; *p < 0.05; one-way ANOVA + Bonferroni's multiple comparison test for **(A-C)**.

The release of superoxide radicals in VKORC1L1-downregulated HCASMC was measured by L-012 chemiluminescence and DCFDA assays. Figures 3A,B illustrate that vascular superoxide release was significantly increased in HCASMC after transfection with VKORC1L1-siRNA (L-012: 186.3 ± 22.62 % vs. scrambled siRNA *p* = 0.009, Figure 3A; DCFDA: 128.7 ± 10.82 % vs. scrambled siRNA, *p* = 0.04, Figure 3B). In order to investigate, if the generation of a specific oxidant is increased upon VKORC1L1 knockdown, we measured H_2_O_2_ generation by an Amplex^TM^ Red Assay. Knockdown of VKORC1L1 by siRNA resulted in slightly, but significantly increased H_2_O_2_ generation in HCASMC (Scrambled siRNA: 100 ± 3.68 % vs. VKORC1L1-siRNA: 105 % ± 3.89 *p* = 0.046, Figure 3C). Thus, VKORC1L1- induced anti-oxidative effects are at least partly mediated by controlling H_2_O_2_ generation.

### VKORC1L1 Regulates Vascular Inflammation in HCASMC

ROS formation and inflammation are closely connected with each other in the pathogenesis of cardiovascular diseases, including atherosclerosis. A pathological increase in ROS induces pro-inflammatory pathways including NF-κB signalling (28, 29). Consequently, we next investigated, if VKORC1L1-knockdown also results in increased inflammatory signalling.

**Figure 4:**
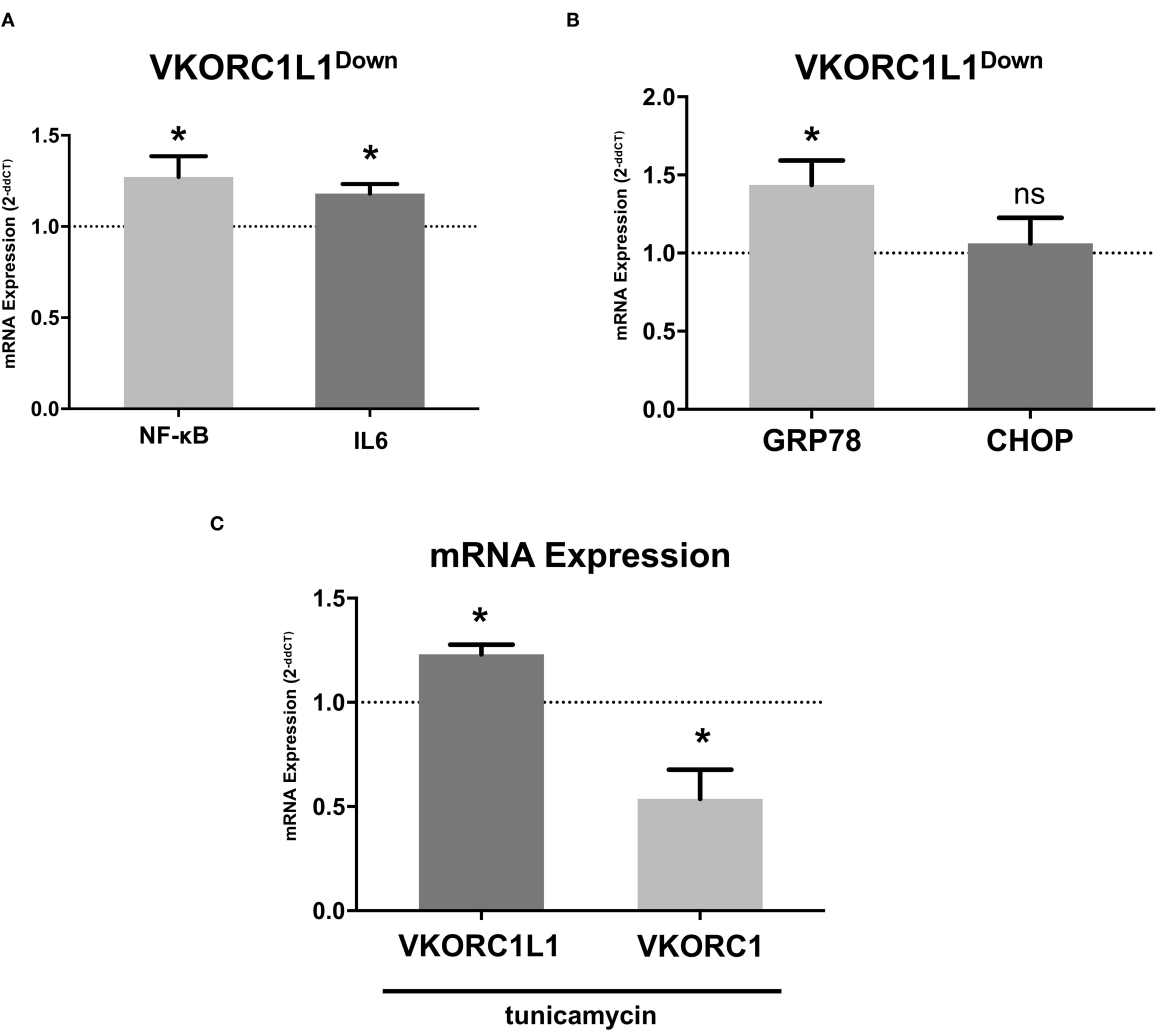
VKORC1L1 regulates vascular inflammation. **(A)** mRNA expression of the pro-inflammatory markers NF-κB and IL-6 in HCASMC after transfection with siRNAs directed against *VKORC1L1* or a scrambled control. Control mRNA expression (=1) is shown as a dotted line. mRNA expression was measured by qPCR and quantified by the 2^−*ddCT*^ method. *n* = 3–8. **(B)** mRNA expression of ER stress markers GRP78 and CHOP in HCASMC after transfection with siRNAs directed against *VKORC1L1* or a scrambled control. Control expression (=1) is shown as a dotted line. mRNA expression was measured by qPCR and quantified by the 2^−*ddCT*^ method. *n* = 4–8. **(C)** mRNA expression of *VKORC1L1* and *VKORC1* in HCASMC after treatment with the ER stress inductor, tunicamycin (6 h, 5 μg/ml). Control expression (DMSO treatment, =1) is shown as a dotted line. *n* = 4. Data are presented as the mean ± SEM; *p < 0.05. Unpaired *t*-test for **(A-C)**.

To study the effect of VKOR1L1 on vascular inflammation, we measured the mRNA expression of the pro-inflammatory markers NF-κB and IL-6 by RT-PCR after transfection of siRNAs directed against VKORC1L1 or a scrambled control sequence. VKORC1L1 downregulation increased the expression of NF-κB mRNA (1.27-fold ± 0.32 vs. control, p = 0.04) and IL-6 (1.18-fold ± 0.12 vs. control, *p* = 0.02, Figure 4A).

Furthermore, VKORC1L1-downregulated HCASMC showed increased mRNA expression levels of GRP78, the main ER stress regulator (1.44- fold ± 0.44 vs. control, *p* = 0.027, Figure 4B). However, mRNA expression of CHOP, another ER Stress protein, was unaltered (1.06- fold ± 0.43 vs. control, *p* = 0.71, Figure 4B). Additionally, stimulation with the ER stress inducer tunicamycin (6 h, 5 μg/ml) promoted mRNA expression of VKORC1L1 while decreasing mRNA expression of VKORC1 (1.23- fold ± 0.09 vs. 0.54- fold ± 0.24, *p* = 0.029, Figure 4C). Thus, VKORC1L1 appears to play a critical role in the unfolded protein response (UPR) and ER stress.

### Menaquinone-7 (MK7) Alleviates HCASMC Remodeling, Inflammation and ER Stress

MK7 is the most potent K-vitamin in humans and is discussed to exert protective properties on progression of cardiovascular disease (5, 30). Therefore, we sought to investigate its role on VSMC remodeling, inflammation and ER Stress *in vitro*.

**Figure 5:**
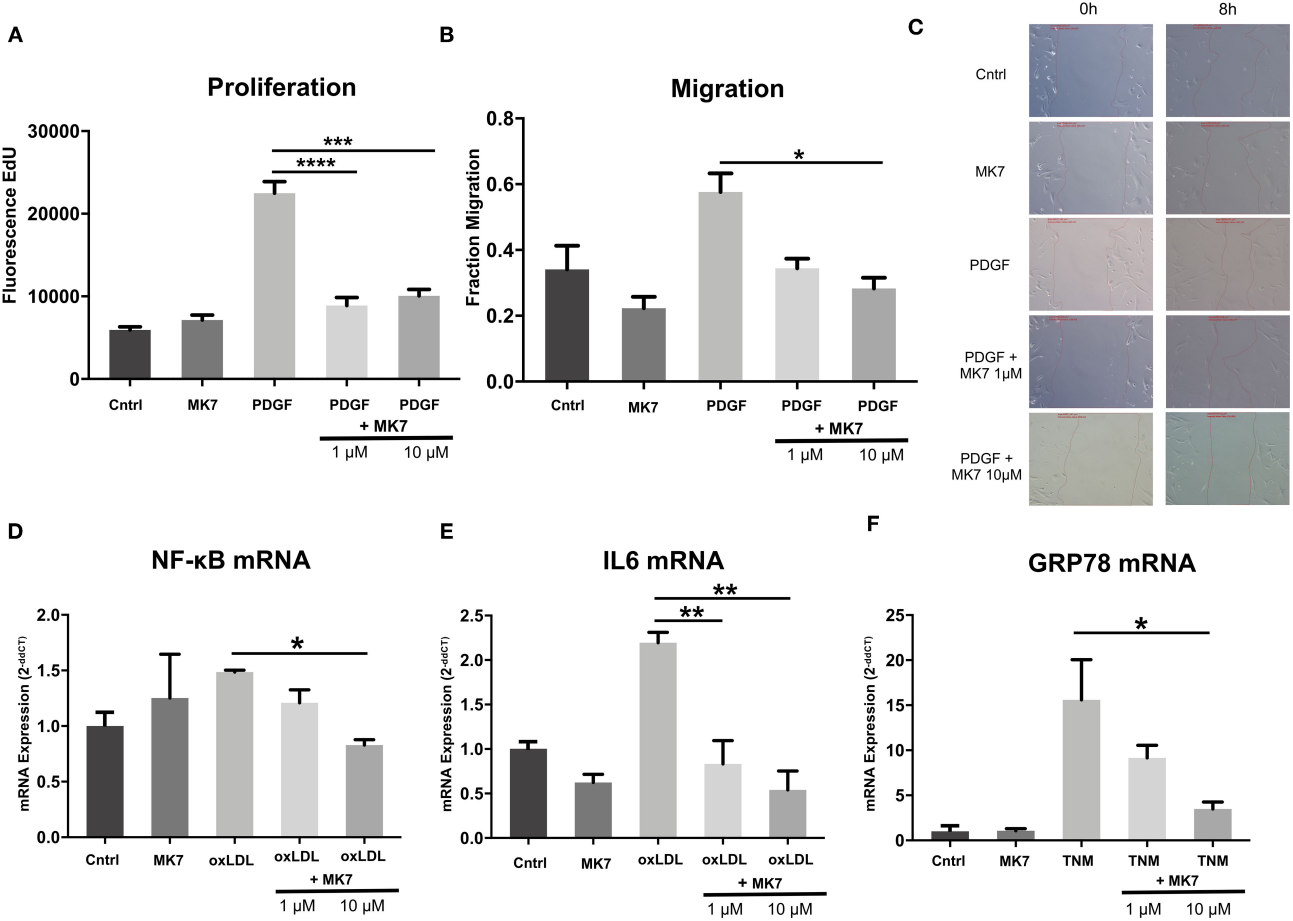
Menaquinone-7 (MK7) alleviates HCASMC remodeling, inflammation and ER stress. **(A)** Proliferation of HCASMC untreated, treated with MK7 (1 μM, 24 h), PDGF (30 ng/ml, 24 h) or PDGF + MK7 (1 μM or 10 μM, 24 h). Measured by an EdU fluorescence assay. *n* = 5. (**B)** Migration of HCASMC after a wound–scratch assay, measured by microscopic visualization after 12 h. Cells treated with MK7 (1 μM, 24 h), PDGF (30 ng/ml, 24 h) or PDGF + MK7 (1 μM or 10 μM, 24 h). *n* = 4. **(C)** Representative pictures of cell migration 0 and 8 h after scratch assay. **(D, E)** mRNA expression of the pro-inflammatory markers NF-κB and IL-6 in HCASMC. Cells treated with MK7 (1 μM, 24 h), oxLDL (25 ng/ml, 24 h) or oxLDL + MK7 (1 μM or 10 μM, 24 h). RNA expression was measured by using qPCR and quantified by the 2^−*ddCT*^ method. n = 3–4. **(F)** mRNA expression of the ER stress marker GRP78 in HCASMC. Cells were treated with MK7 (1 μM, 24 h), tunicamycin (TNM, 5 μg/ml, 6 h) or TNM + MK7 (1 μM or 10 μM, 24 h). RNA expression was measured by using qPCR and quantified by the 2^−*ddCT*^ method. *n* = 3. Data are presented as the mean ± SEM; *p < 0.05, **p < 0.01, ***p < 0.001, ****p < 0.0001. One-way ANOVA + Bonferroni's multiple comparison test for **(A-E)**.

HCASMC were treated with DMSO (control), treated with MK7 (1 μM, 24 h), PDGF (30 ng/ml, 24 h) or with PDGF (30 ng/ml, 24 h) and MK7 (1μM or 10 μM, 24 h). Proliferation was measured by EdU-Fluorescency after 24 h. As expected, PDGF treatment increased HCASMC proliferation. Intriguingly, co-Treatment with MK7 attenuated PDGF-induced proliferation (Fluorescence signal: PDGF: 22495 vs. PDGF+MK7 1 μM: 8897, *p* = 0.0008; PDGF: 22495 vs. PDGF+MK7 1 μM: 10074, *p* = 0.0018, Figure 5A).

Next, HCASMC were treated accordingly and a wound scratch assay was performed. Migration of HCASMC visualized after 12 h showed that PDGF, as expected, induced migration of HCASMC and co-treatment with higher concentrated MK7 reduced PDGF-induced migration (Fraction Migration: PDGF: 0.57 vs. PDGF+MK7 10 μM: 0.28, *p* = 0.04, Figures 5B,C).

Next, we tested the effect of MK7 on mRNA levels of vascular inflammation markers (Figures 5D,E). Therefore, HCASMC were treated with DMSO (control), treated with MK7 (1μM, 24 h), oxLDL (25 ng/ml, 24 h) or oxLDL and MK7 (1μM or 10 μM, 24 h).

As expected, oxLDL treatment increased expression of NF-κB (1.49-fold ± 0.03) and IL-6 (2.2-fold ± 0.17). When co-treating with MK7, IL-6 expression was reduced at both of the used MK7 concentrations (oxLDL + MK7 1 μM: 0.83-fold, *p* = 0.004 vs. oxLDL only; oxLDL + MK7 10 μM: 0.54-fold ± 0.36, *p* = 0.001 vs. oxLDL only) while higher concentration of MK7 abrogated NF-κB expression (oxLDL + MK7 10 μM: 0.82-fold, *p* = 0.019 vs. oxLDL only).

Finally, we analysed the effect of MK7 on ER Stress induction (Figure 5F). HCASMC were untreated, treated with MK7 (1μM, 24 h), tunicamycin (5 μg/ml, 6 h) or tunicamycin and MK7 (1μM or 10 μM, 24 h). Tunicamycin treatment led to highly increased mRNA expression of GRP78 (15.6-fold ± 7.7). Co-treatment with 10 μM MK7 reduced tunicamycin-induced GRP78 expression (3.5-fold ± 1.3, *p* = 0.015 vs. tunicamycin only).

## Discussion

In this study, we investigated for the first time the role of the VKOR isoenzyme VKORC1L1 in vascular biology. We demonstrated that VKA-promoted adverse remodeling is accompanied by a downregulation of VKORC1L1 *in vivo*. *In vitro*, we showed that VKORC1L1 downregulation promotes maladaptive remodeling and inflammation in vascular smooth muscle cells. Moreover, we describe a novel connection between the vitamin K cycle and oxidative protein folding. VKORC1L1 downregulation promotes ER stress, a major driver of vascular inflammation. Finally, we present data showing that the vitamin K derivative menaquinone-7 is able to alleviate remodeling, inflammation and ER stress in VSMC.

Therapeutic anticoagulation with VKA has been described to be associated with progressive vascular calcification and vulnerable atherosclerotic plaques in both mice and humans (10, 31, 32). While the role of VKA in calcification by way of the matrix Gla-protein pathway has been extensively studied, further mechanisms of VKA-induced cardiovascular disease remain to be elucidated. Although warfarin intake is known to exert a complex influence on the immune system (33), the role of warfarin in vascular inflammation and anti-oxidation has not yet been resolved.

By utilising a widely used carotid-artery injury model, we could confirm that VKA promotes maladaptive vascular remodeling in mice. Recent studies suggested that the VKOR isoenzyme VKORC1L1 has anti-oxidative properties and is less sensitive to VKA than VKORC1 (18, 19). In our injury model, two-week long VKA treatment lead to reduced aortic *VKORC1L1* mRNA. *In vitro* experiments confirmed warfarin treatment to reduce VKORC1L1 expression in VSMC. Our *in vitro* results hint at an involvement of VKORC1L1-downregulation in warfarin-induced vascular damage. Therefore, the inhibition of VKORC1L1 appears to be a plausible driver of neointima formation and restenosis.

Westhofen et al. demonstrated an increase in ROS formation of HEK-293 cells after VKORC1L1-downregulation via siRNAs (18). We confirmed these findings in HCASMC, suggesting that VKORC1L1 regulates mechanisms of anti-oxidation in the vasculature.

NF-κB is a transcription factor regarded as one of the key regulators of inflammatory processes, including cardiovascular inflammation (34). We showed that siRNA-mediated VKORC1L1-downregulation increases NF-κB expression in HCASMC. Long-term oral intake of warfarin was previously described to be associated with increased IL-6 production in rats (35). In our study, we found that VKORC1L1-downregulation increased the mRNA expression of IL-6. Warfarin-induced vascular inflammation may therefore be at least partly mediated by VKORC1L1 and the NF-κB/IL-6 pathway.

The cellular localization of the VKOR proteins and the effect that VKORC1L1 has on anti-oxidation and inflammation strongly suggest that there is a connection to the UPR. Multiple studies have described that VKOR proteins interact with protein disulfide-isomerases (23, 36). Herein, we present data linking VKORC1L1 to the UPR and ER stress pathways. After VKORC1L1-downregulation, GRP78, a marker for general UPR activation, was found to be increased while CHOP expression remained unchanged. CHOP is mainly activated by the PERK/eIF2α/ATF4 pathway, while downstream activation of GRP78 is induced by the ATF6 pathway (37, 38). Hence, specific UPR pathways are activated upon VKORC1L1 inhibition. Our results extend and support recent data of *Furmanik et al*., who showed that warfarin induces GRP78 expression and subsequent vascular calcification (39).

Finally, after describing putative mechanisms for VKA-induced vascular dysfunction, we found also that MK7 treatment can dose-dependently reduce vascular remodeling, inflammation and ER stress *in vitro*. K2 vitamins have previously been attributed anti-inflammatory effects (40, 41). However, we show here for the first time that vitamin K can alleviate inflammation in vascular cells. As far as we are aware, this is also the first evidence for vitamin K-promoted repression of ER stress in cells of any type. A possible mechanism underlying the anti-oxidative effects of vitamin K might be due to a vitamin K-induced increasement in VKORC1L1 activity. The increasement in VKORC1L1 activity could then dampen oxidative stress and subsequent inflammation. Further studies are warranted to elucidate these mechanisms.

In conclusion, we found that VKA promote neointima formation and that this is at least partly mediated by VKORC1L1 inhibition. Contrarily, MK7 supplementation reduces aberrant proliferation and inflammation in VSMC. Due to its favourable risk profile, MK7 may represent a feasible therapeutic target to reduce neointima formation. Main limitation of our study are the limited *in vivo* read-outs. In this study, we mainly focussed on the *in vitro* functions of VKORC1L1 on VSMC. Future studies utilising VKORC1L1^−/−^ and VKORC1L1/ApoE^−/−^ mice are currently ongoing and will further elucidate the role of VKORC1L1 during vascular injury and its connection to ER Stress. Additionally we did not yet investigate the presumed *in vivo* effects of MK7 on inflammation and ER Stress. The association of MK7 and VKORC1L1-deficiency will also be subject of upcoming projects. These results will improve our understanding of the manifold effects of vitamin K on the cardiovascular system.

## Data Availability Statement

The raw data supporting the conclusions of this article will be made available by the authors, without undue reservation.

## Ethics Statement

The animal study was reviewed and approved by National Office for Nature, Environment, and Consumer Protection in Recklinghausen, North Rhine-Westphalia (Landesamt für Natur, Umwelt und Verbraucherschutz; LANUV.

## Author Contributions

AA, MA, CM, JO, GN, and VT done conceptualization AA, MA, and VT worked on the methodology. AA, MA, ER, MB, and SZ done formal analysis and investigation. AA, MA, and VT wrote and prepared the original draft. All authors reviewed and edited the manuscript. AA, GN, and VT done funding acquisition. GN and VT provided the resources and supervised the work.

## Funding

This work was supported by the medical faculty of the University of Bonn (BONFOR Grant No. 2018-1A-02 to AA). ER was supported by the German Heart Foundation (Kaltenbach Scholarship).

## Conflict of Interest

The authors declare that the research was conducted in the absence of any commercial or financial relationships that could be construed as a potential conflict of interest.

## Publisher's Note

All claims expressed in this article are solely those of the authors and do not necessarily represent those of their affiliated organizations, or those of the publisher, the editors and the reviewers. Any product that may be evaluated in this article, or claim that may be made by its manufacturer, is not guaranteed or endorsed by the publisher.

## Abbreviations

CHOP: C/EBP homologous protein
DCFDA, 2': 7'-dichlorofluorescein diacetate
ER: Endoplasmic reticulum
GRP78: Glucose-regulated protein 78kDa
HCASMC: Human coronary artery smooth muscle cells
IL-6: Interleukin 6
MK7: Menaquinone-7
NFκB: Nuclear factor ‘kappa-light-chain-enhancer’ of activated B-cells
oxLDL: Oxidized low-density lipoprotein
PDGF: Platelet-derived growth factor
ROS: Reactive oxygen species
siRNA: Small interfering RNA
VKA: Vitamin K antagonists
VKORC1: Vitamin K epoxide reductase complex subunit 1
VKORC1L1: Vitamin K epoxide reductase complex subunit 1-like 1
VSMC: Vascular smooth muscle cells.
