# Supplementary material for: Vitamin K Epoxide Reductase Complex Subunit 1-Like 1 (VKORC1L1) Inhibition Induces a Proliferative and Pro-inflammatory Vascular Smooth Muscle Cell Phenotype
Source: Front Cardiovasc Med. 2021 Oct 27;8:708946. doi: 10.3389/fcvm.2021.708946 (PMC8578699; doi:10.3389/fcvm.2021.708946)
Supplement: Supplementary file 1 [file Data_Sheet_1.docx]

**Contribution to the Field:**

Atherosclerosis is the underlying pathology that causes coronary artery disease, myocardial infarction, stroke and peripheral vascular disease. Collectively, these conditions are among the most prevalent causes of death worldwide. Extensive proliferation of vascular smooth muscle cells (VSMC) is a common hallmark of atherosclerosis and promotes restenosis after stenting.

Vitamin K is mostly known for its role in activation of coagulation factors, but vitamin K intake is also associated with a lower risk for coronary artery disease and cardiovascular death. Contrarily, therapeutic anticoagulation with vitamin K antagonists was frequently ascribed an adverse effect on atherosclerosis progression. The recently described Vitamin K epoxide reductase complex subunit 1-like 1 (VKORC1L1) is involved in vitamin K maintenance and exerts antioxidant properties. In this study, we found that treatment with vitamin K antagonists promotes maladaptive neointima formation and reduces VKORC1L1 expression *in vivo*. Utilizing *in vitro* models, we elucidated previously unknown protective properties of VKORC1L1 in VSMC biology. Finally, we demonstrated that treatment with vitamin K2 preserves VSMC function by reducing aberrant proliferation, migration and inflammation.

Our study introduces a novel, protective target in vascular biology with a high potential for clinical translation.

**Supplementary Figure 1:** siRNA Transfection against VKORC1L1

**(A)** VKORC1L1 mRNA expression in HCASMC after siRNA Transfection directed against VKORC1L1 or scrambled control. Expression measured by qPCR and quantified by 2^^-ddCT^ Method. n = 3, Data are presented as the mean ± SEM; *p < 0.05. Unpaired t-test.
