# Supplementary figures and images for: Vitamin K Epoxide Reductase Complex Subunit 1-Like 1 (VKORC1L1) Inhibition Induces a Proliferative and Pro-inflammatory Vascular Smooth Muscle Cell Phenotype
Source: Front Cardiovasc Med. 2021 Oct 27;8:708946. doi: 10.3389/fcvm.2021.708946 (PMC8578699; doi:10.3389/fcvm.2021.708946)

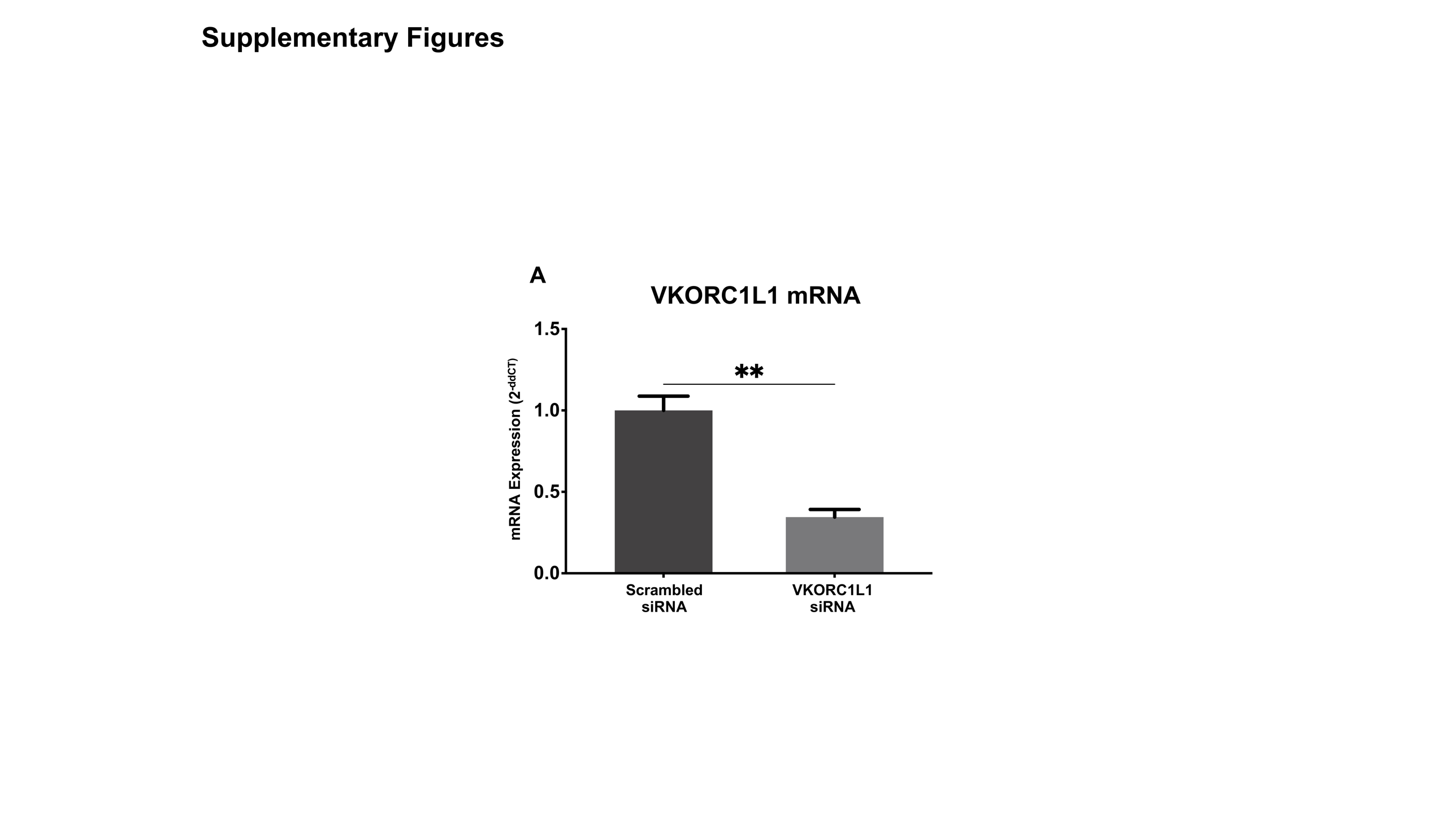

Supplement: Supplementary file 2 [file Image_1.JPEG]
